# Effects of High Pressure on In Vitro Bioavailability of Curcumin Loaded in Whey Protein Isolate/Carrageenan Composite Emulsion Gel: In Vitro Digestion Coupled with Cell Culture Model

**DOI:** 10.3390/foods13233782

**Published:** 2024-11-25

**Authors:** Jiayue Zhao, Xinmeng Zhang, Yanan Huang, Yan Tan, Shuang Ren, Fang Yuan

**Affiliations:** 1 Key Laboratory of Healthy Beverages, China National Light Industry Council, College of Food Science & Nutritional Engineering, China Agricultural University, Beijing 100083, China; 2 Department of Food Science, University of Tennessee, Knoxville, TN 37996, USA

**Keywords:** high pressure processing, curcumin, emulsion gel, in vitro digestion, Caco-2 cells

## Abstract

The oral bioavailability of curcumin is inherently low, which significantly limits its application in food systems. The objective of this study was to evaluate the impact of high-pressure processing on the stability and bioaccessibility of curcumin within an emulsion gel during simulated gastrointestinal transit and to assess its cellular uptake. Our findings suggest that increasing pressure levels and high κ-carrageenan concentrations can enhance the stability of the curcumin delivery system. Elevated κ-CG concentrations were found to retard the action of proteases on dissociating protein molecules from the gel network. The emulsion gel effectively slowed the release of free fatty acids and reduced the curcumin release rate during the gastric phase. Scanning electron microscopy images revealed that higher pressures induced the formation of a more uniform and dense network structure in the gel. While the gel network structures were well-preserved after gastric digestion, they were disrupted into smaller particles following intestinal digestion, with particle size increasing with higher applied pressures. Cytotoxicity assays indicated that the digesta from the intestinal phase was highly toxic to Caco-2 cells. Among the tested samples, the emulsion gel prepared with 1.0% κ-CG at 600 MPa demonstrated the highest curcumin bioavailability, reaching 63.82 ± 7.10%. These findings underscore the potential of HPP-induced emulsion gels as a viable delivery system for enhancing curcumin bioaccessibility and cellular uptake.

## 1. Introduction

Chronic diseases, including cardiovascular diseases, metabolic syndrome, and cancer, pose a substantial global health challenge, with their prevalence increasing at an alarming rate [1,2,3]. The exploration of natural food-derived compounds as potential mitigants of these conditions has become a focal point of interest in clinical research [4,5].

Curcumin (CUR), a polyphenolic compound extracted from Curcuma longa [6], has been employed in the treatment of a range of conditions, including coronavirus [7,8], cardiovascular disease [9,10], arthritis [11], and cancer [12]. The diverse biological activities of this substance, including anti-inflammatory, antioxidant, antiviral, antibacterial, and antitumor effects, are thought to underpin its health benefits [13,14]. However, the clinical application of curcumin is limited by its high melting point, low solubility, and poor oral bioavailability, particularly due to its hydrophobic nature, which impedes absorption by the small intestine’s epithelial cells [15]. The encapsulation of curcumin has emerged as a promising method to enhance its oral bioavailability, with the utilization of a range of delivery systems, including nanostructured lipid carriers [16], nanoparticles [17], micelles [18], emulsion [19], colloid [20], being investigated to improve its dispersion, stability, and bioavailability.

Emulsion gels, as semi-solid systems with a gel network, provide an effective platform for encapsulating fat-soluble substances, offering stabilization and controlled release of fat-soluble active ingredients [21,22]. They have become an ideal vehicle for bioactive compounds, providing enhanced stability and safety over traditional emulsions. Research indicates that emulsion gels are effective in encapsulating lycopene [23], allicin [24], and astaxanthin [25], thereby enhancing their stability and bioaccessibility and facilitating their targeted release. While thermal, enzyme, ion, and acid induction methods have been widely studied for emulsion gel preparation, ultra-high pressure (UHP) processing has not been fully explored. UHP technology, characterized by its high efficiency, low energy consumption, absence of impurity introduction, and preservation of original flavors, offers significant potential for emulsion gel production. 

Emulsion gels can be categorized based on their matrix into protein, polysaccharide, and mixed emulsion gels [26]. Polysaccharides, which are typically resistant to digestive enzymes within the stomach and small intestine yet susceptible to degradation by colonic microorganisms, represent an optimal choice for targeted delivery systems [27,28]. The combination of proteins with polysaccharides, such as inulin, κ-carrageenan, and konjac glucomannan, can resist enzymatic hydrolysis and delay the release of functional factors in the stomach and small intestine [29]. Proteins and polysaccharides can synergistically stabilize the oil–water interface in emulsion systems and form high-performance composite gels in the continuous phase. Whey protein isolate (WPI), a premium protein extracted from dairy, is known for its essential amino acid profile and bioactive constituent content. Its amphiphilic nature and ability to emulsify hydrophobic groups render it an effective emulsifier. Research has confirmed that the inclusion of WPI significantly bolsters the firmness of emulsion gels [30,31]. κ-Carrageenan (κ-CG), which contains a high ratio of anionic sulfuric acid groups, can form complexes with WPI, promoting interactions between unfolded protein molecules under high pressure and enhancing the strength of the resulting gel.

Current research on UHP-induced emulsion gels has primarily focused on their physicochemical properties, with scant attention given to their potential for enhancing the uptake of hydrophobic bioactives. This study aimed to develop a UHP-induced emulsion gel delivery system to improve the dispersion, stability, and bioavailability of curcumin. We evaluated the gastrointestinal transit of curcumin encapsulated in WPI/κ-CG composite emulsion gels under varying pressures and holding times using gastrointestinal modeling. Additionally, we investigated curcumin absorption in human epithelial adenocarcinoma (Caco-2) cells to elucidate its bioavailability. The findings of this study are crucial for the advancement of more efficacious delivery systems for hydrophobic bioactives, potentially offering safer and more efficacious dietary interventions for chronic disease patients, thereby improving their quality of life.

## 2. Materials and Methods

### 2.1. Materials

WPI was purchased from Davisco Foods International Inc. in Savage, MN, USA (purity > 95%). κ-CG was obtained from CPKelco in Atlanta, GA, USA (purity > 90%). CUR was acquired from China National Pharmaceutical Group Co., Ltd. (Shanghai, China) (purity > 97%). Medium-chain triglycerides (MCT) were purchased from Musim Mas Co., Ltd. (Singapore) (purity > 99%). All enzymes used in vitro-simulated digestion were of analytical grade and obtained from Sigma-Aldrich (Wicklow, Ireland). The Caco-2 cell line was purchased from the ATCC cell bank (Gaithersburg, MD, USA). The reagents used in the cell culture medium were purchased from GIBCO (New York, NY, USA). All other chemical agents were analytical grade and obtained from Beijing Chemical Plant Co., Ltd. (Beijing, China). Water purified by a Milli-Q system (Millipore, MA, USA) was used for all the experiments.

### 2.2. Preparation of HPP-Induced WPI/κ-CG Composite Emulsion Gel Loaded with CUR

#### 2.2.1. Preparation of WPI/κ-CG Compound Solution

We formulated a 24% *w*/*v* Whey Protein Isolate (WPI) solution by measuring out the WPI powder, dissolving it in deionized water, and then mixing it with a magnetic stirrer at ambient temperature until the WPI was fully dissolved. For the κ-carrageenan (κ-CG) solution, we prepared concentrations of 1.6%, 2.0%, and 2.4% *w*/*v* by dispersing the powder in deionized water and stirring until complete dissolution. Subsequently, the WPI solution was combined with the κ-CG solution in a 1:1 volume ratio. This resulted in a final mixture with a WPI concentration of 12% *w*/*v* and κ-CG concentrations of 0.8%, 1.0%, and 1.2% *w*/*v*. The combined solution was continuously stirred overnight at room temperature, and the pH was adjusted to neutrality (7) using a 1 mol/L sodium hydroxide solution. Finally, 0.02 wt% sodium azide was incorporated as a preservative to prevent microbial growth.

#### 2.2.2. Formulation of CUR-Enriched O/W Emulsion

Following a modified approach from Ma et al. [32], curcumin was dissolved in MCT. Initially, 1 g of curcumin was mixed with 200 g of MCT and stirred magnetically in darkness for 10 min, followed by ultrasonication at 405 W with 1-s intervals for 30 min. The mixture was then centrifuged at 3000 rpm for 5 min to eliminate undissolved particles. The supernatant was retained and kept at 25 °C in darkness for subsequent use. A measured amount of CUR-laden MCT was incorporated into the prepped WPI/κ-CG blend to achieve a 30% oil phase (*w*/*w*). The mixture was emulsified at 10,000 rpm for 3 min using a T25 disperser (IKA, Staufen, Germany) to form a coarse emulsion, which was then refined through high-pressure homogenization (NS1001L2K, Niro-Soavi, Parma, Italy) at 50 MPa for three cycles to yield a fine emulsion.

#### 2.2.3. High-Pressure Treatment

The emulsion was cooled to ambient temperature and transferred into a 50 mL plastic centrifuge tube, vacuum-sealed in a polyethylene bag. High-pressure processing (HPP) was conducted using a laboratory-scale press (L2-700/1, Tianjin Huatai Senmiao Biological Engineering Technology Ltd., Tianjin, China) with water as the pressure medium. The experimental pressures were set at 400 MPa, 500 MPa, and 600 MPa, and the holding times were 10, 20, and 30 min, respectively. The rate of pressure rise was about 6.5 MPa/s, and the rate of release was about 20 MPa/s. Various parameters of the gels were measured after the treated samples were stored in a refrigerator at 4 °C for 24 h.

### 2.3. In Vitro Static Simulated Digestion

The emulsion gels underwent a static in vitro digestion mimicking the human upper gastrointestinal tract, adhering to the INFOGEST protocol [33] with minor adjustments. For the gastric phase, 1 g of gel was combined with 10 mL of SGF (3.2 mg/mL pepsin, 0.2% NaCl), and the pH was adjusted to 1.5 with 1 M HCl. The mixture was incubated at 37 °C for 2 h with agitation. Afterward, the pH was raised to 6.5 with 1 M NaOH, and 30 mL of SIF (0.68% KH_2_PO_4_, 10 mg/mL bile salts, 3.2 mg/mL pancreatin, 0.4 mg/mL lipase) was added. The pH was readjusted to 7.0, and the mixture was further incubated at 37 °C for 2 h. Digestion was halted using an ice bath, and the micellar fraction was isolated by centrifugation at 3000 rpm for 15 min at 4 °C. Samples were stored at −20 °C for future analysis. 

### 2.4. Size Distribution

The size distribution in the gastric and intestinal phases was analyzed via dynamic light scattering (DLS) using a Zetasizer Nano-ZS90 (Malvern Instruments, Worcestershire, UK). Samples were diluted 10-fold and measured at a 90° angle after a 60-s equilibration period. 

### 2.5. Free Fatty Acid Release 

The small intestine phase digesta were centrifuged at 14,000 rpm for 20 min at 4 °C to isolate the micelle phase. Free fatty acid (FFA) content was determined using a colorimetric kit (Nanjing Jiancheng Technology Co., Ltd., Nanjing, China, #A042-1, based on the formation of copper-FFA complexes.

### 2.6. Free Amino Acid Release

Free amino acid (FAA) content was assessed using the ninhydrin method. Digesta were mixed with 5% trichloroacetic acid, incubated, centrifuged, and the supernatant was analyzed for FAA after dilution. Absorbance was measured at 426 nm using a UV1800 spectrophotometer (Shimadzu Corporation, Kyoto, Japan), and FAA levels were determined from a standard curve.

### 2.7. Curcumin Release Rate

Curcumin release was quantified by mixing 1 mL of micelle phase with 4 mL ethanol, centrifuging, and measuring the supernatant’s absorbance at 426 nm. Curcumin concentration was determined using a standard curve, and release efficiency was calculated as

(1) $$release\;rate\;(\%)=\frac{C_{micelle}}{C_{digest}}\times 100\%$$

where C_micelle_ is the curcumin concentration in micelle fraction, and C_digest_ is the curcumin concentration in total digesta.

### 2.8. Microstructure

SEM (SU8010, Hitachi, Tokyo, Japan) was used to examine the microstructure of emulsion gels and digesta after lyophilization and gold sputter-coating. Images were captured at 3.0 kV and 20.0 k magnification.

### 2.9. Cell-Based Assays

#### 2.9.1. Cell Monolayer Model

Caco-2 cells were cultured in DMEM with supplements and used between passages 40–60. For monolayer formation in 12-well plates, cells were seeded at 1.2 × 10^5^ cm^−2^, and the medium was refreshed every 3–4 days. Experiments were conducted 21–25 days post-seeding.

#### 2.9.2. Cytotoxicity Assay

Cytotoxicity was evaluated using the MTT assay. Caco-2 cells were seeded in 96-well plates and exposed to digesta. After incubation and MTT staining, absorbance was measured at 490 nm, and viability was expressed as a percentage of control absorbance.

#### 2.9.3. Cellular Curcumin Uptake

Curcumin uptake by Caco-2 monolayers was determined after exposure to micellar phase digesta. Cell integrity was monitored, and curcumin concentration in the basolateral medium was analyzed against a standard curve. Bioavailability was calculated as

(2) $$bioavailability\;(\%)=\frac{C_{BL}}{C_{AP}}\times 100\%$$

where C_BL_ is the curcumin concentration in the basolateral medium, and C_AP_ is the curcumin concentration in the apical medium.

### 2.10. Statistical Analysis

Data were analyzed in triplicate using SPSS 22.0, with results presented as mean ± SD. ANOVA with Duncan’s test was used to assess statistical significance at *p* < 0.05.

## 3. Results and Discussion

### 3.1. Size Distribution

**Figure 1:**
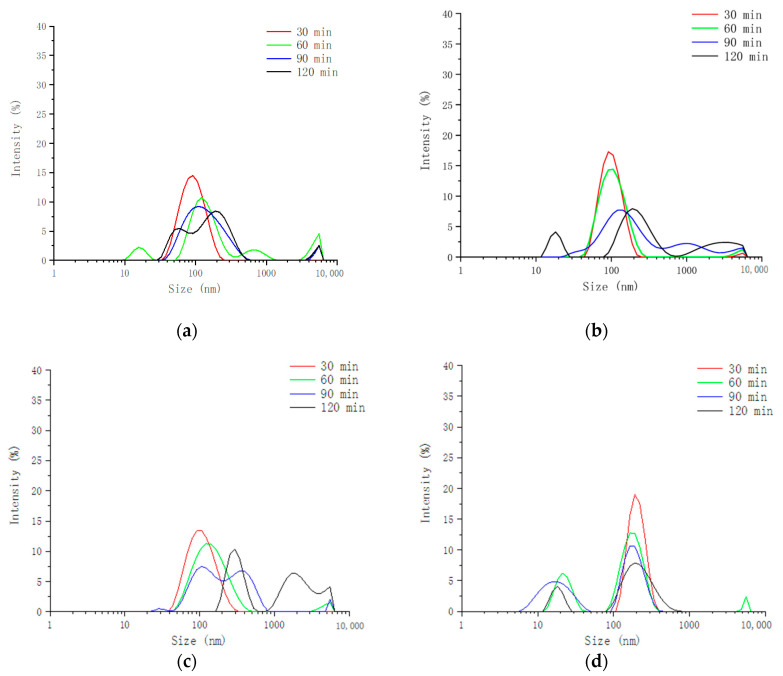

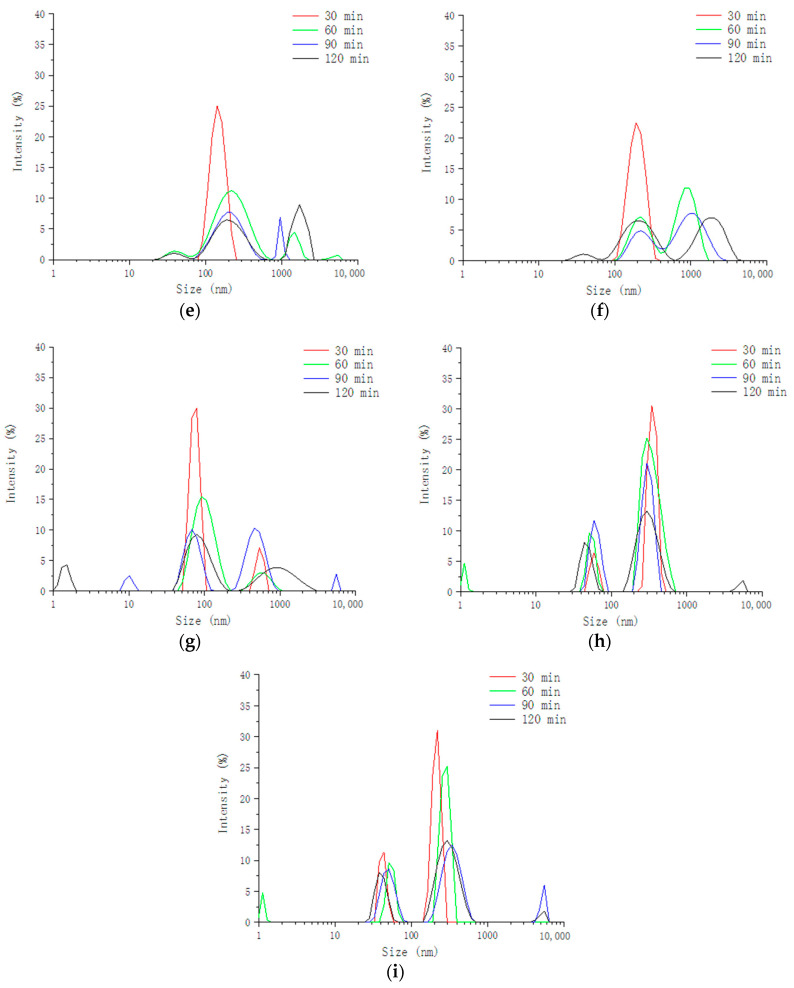
Size distribution of digesta from simulated intestinal phase after different digestion times. (**a**) 0.8% κ-CG 400 MPa; (**b**) 0.8% κ-CG 500 MPa; (**c**) 0.8% κ-CG 600 MPa; (**d**) 1.0% κ-CG 400 MPa; (**e**) 1.0% κ-CG 500 MPa; (**f**) 1.0% κ-CG 600 MPa; (**g**) 1.2% κ-CG 400 MPa; (**h**) 1.2% κ-CG 500 MPa; (**i**) 1.2% κ-CG 600 MPa.

Figure 1 shows the size distribution of the digesta of emulsion gels after different digestion times. The data in the gastric digestive fluid we put in the Appendix A. As shown in the figure, in gastric digesta, with the increase in digestion time, the range of size distribution became wider and wider, indicating that the types and sizes of droplets in digesta were becoming more and more diverse. This might be due to the gel structure being gradually destroyed by pepsin. The interface structure of oil droplets wrapped by protein and polysaccharide was destroyed and destabilized, leading to flocculation and coalescence of droplets; on the other hand, the completely released oil droplets may be stabilized by the polypeptide produced by WPI, resulting in droplets of smaller sizes. Compared with the gastric digesta, the wider size distribution in the intestinal digesta is mainly due to the fact that in the process of oil hydrolysis, curcumin is continuously released from the oil and loaded into the mixed micelles or vesicles formed by the self-assembly of bile salts, free fatty acids, and monoglycerides, resulting in droplets of smaller sizes in the system.

However, HPP had no noticeable effect on the size distribution of intestinal digesta. This may be because the intestinal digestion stage mainly involves hydrolysis of biomacromolecules rather than disruption of the gel structure. With increasing concentration of κ-CG, the size distribution of the digesta became more concentrated, especially the intestinal digesta. It may be because κ-CG cannot be decomposed by enzymes in the human digestive tract; thus, it can stabilize on the oil drop surface for longer. The higher the κ-CG concentration was, the more oil droplets were stabilized by it, which prevented enzymes from contacting the internal lipids and the expansion of size distribution. 

### 3.2. Free Fatty Acid Release

**Figure 2:**
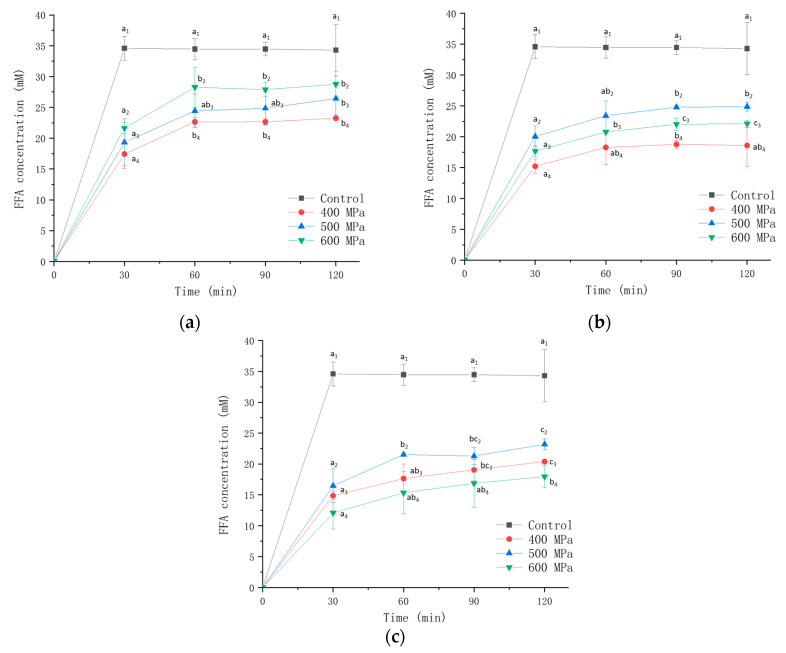
The concentration of FFA in the digestive fluid during intestinal digestion of emulsion gel prepared under different pressures and κ-CG concentrations: (**a**) 0.8% κ-CG; (**b**) 1.0% κ-CG; (**c**) 1.2% κ-CG. Different letters in the same picture indicate a significant difference (*p* < 0.05, *n* = 3).

Figure 2 shows the changes in free fatty acid (FFA) release of the gels prepared at differing pressures and κ-CG concentrations, with an oil–water mixture that was not emulsified and pressurized used as a control. Since the SIF did not contain lipase, this experiment only measured the content of FFA during simulated intestinal digestion. It can be seen that with the increase in digestion time, the FFA concentration of the control group quickly reached the maximum within 30 min and then remained almost unchanged. The authors of [34] also found a similar phenomenon and believed that the rapid release of FFA indicated that bile salts were quickly adsorbed on the oil droplet surface, and then lipase quickly attached to the surface and then hydrolyzed triacylglycerols. And after 30 min, the slower increase in concentration was due to the fact that after most of the triacylglycerols were hydrolyzed, the resulting FFA accumulated on the surface of the oil droplets due to its amphipathy, making it difficult for lipase to access the core triglycerides. Compared to the control group, it could be seen that the formation of emulsion gel slowed down the release of FFA. It could be due to the fact that the remaining large gel fragments after gastric digestion reduced the initial contact area of lipase and lipids, thereby inhibiting lipid digestion.

As can be seen in the figure, when the κ-CG concentration was 0.8%, the amount of FFA released grew as the pressure was added; when the κ-CG concentration was 1.0% or 1.2%, the amount of FFA released first increased as the pressure was added and then decreased, reaching a maximum at 500 MPa. In addition, it can also be observed that the higher the κ-CG concentration, the lower the FFA released. Yao et al. [35] attributed the decrease in lipolysis rate with the increase in polysaccharide concentration to the interaction between polysaccharides and bile salts, lipase, etc. Guo et al. [36] found that when the gellan gum content in the gel increased, the lipolysis rate slowly dropped, which might be due to the more compact structure of the emulsion gel at a larger polysaccharide concentration. Additionally, it was reported that the interfacial membrane formed by macromolecular emulsifiers such as κ-CG could resist the competitive adsorption of bile salts [37]. 

### 3.3. Free Amino Acid Release

**Figure 3:**
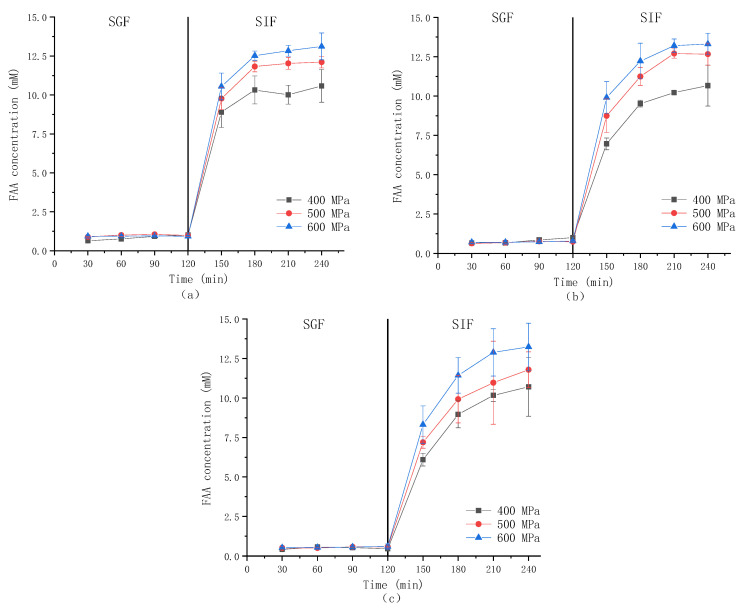
The concentration of FAA in the digestive fluid during digestion of emulsion gel prepared under different pressures and κ-CG concentrations: (**a**) 0.8% κ-CG; (**b**) 1.0% κ-CG; (**c**) 1.2% κ-CG.

Figure 3 shows the changes in the release of FAA during the simulated gastrointestinal digestion of the gel prepared under different pressures and κ-CG concentrations. As the figure shows, the concentration of FAA in gastric digesta was much lower than that in intestinal digesta. It could be ascribed to the fact that, on the one hand, most of the gel structure still existed during gastric digestion, and then, the β-lactoglobulin in WPI was not easily digested by gastric protease but easily digested by trypsin [38]. As the pressure increased, the FAA content increased, indicating that HPP increased the digestibility of WPI in the gel. In previous studies [39,40], the protein’s secondary structure after various pressure treatments was measured, and the results showed that the α-helix and β-sheet content declined as the pressure was raised, indicating that high pressure made the structure of the protein more unstable and easily exposed to enzymes. It was also reported that by compressing the volume of protein molecules, high pressure could change the secondary bonds in the tertiary and quaternary structure of the protein, causing structure stretch and protein depolymerization [41]. As the concentration of κ-CG increased, the increasing trend of FAA became slower, which could be ascribed to the fact that the increase in κ-CG concentration delayed the protein released from the gel structure.

### 3.4. Curcumin Release Rate

**Figure 4:**
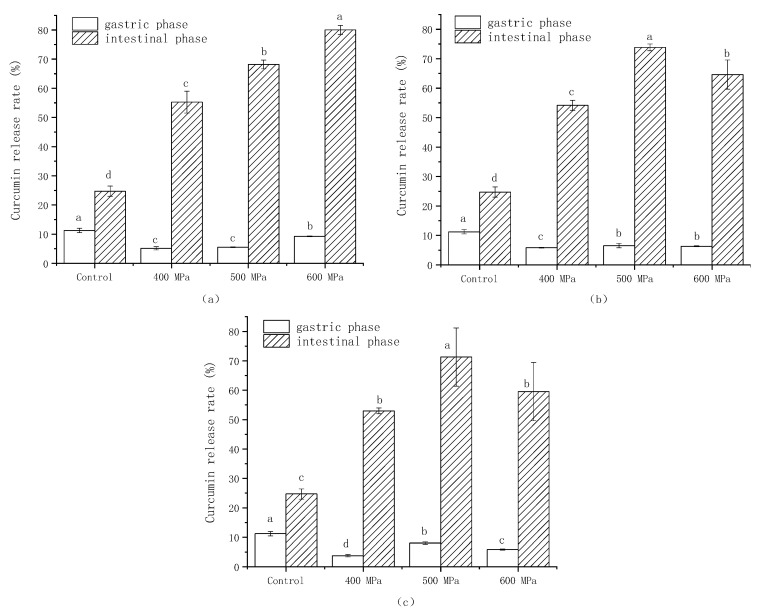
Release rate of curcumin after gastric digestion and intestinal digestion of the gel prepared under different pressures and κ-CG concentrations: (**a**) 0.8% κ-CG; (**b**) 1.0% κ-CG; (**c**) 1.2% κ-CG. Note: Different letters in the same picture indicate a significant difference (*p* < 0.05, *n* = 3).

Figure 4 shows the changes in the curcumin release rate of the gels prepared under various pressures and κ-CG concentrations after simulated gastrointestinal digestion, with an unemulsified oil–water mixture as a control. As the figure shows, the emulsion gel delayed the release of curcumin in the gastric tract and significantly improved the release rate of curcumin in the intestinal tract. According to reports, curcumin has a low absorption rate in the human body due to its lipophilicity. Only when it is successfully transferred to the micellar phase in the intestine can it be transferred through the human intestinal epithelial cells and, finally, enter the blood circulation [42]. In comparison with control, the emulsion gel could inhibit the release rate of curcumin in gastric digestion mainly because it still retained most of the gel structure after gastric digestion, which prevented the curcumin from being released into the digestion fluid. 

At the concentrations of κ-CG of 0.8%, the release rate of curcumin was higher with the pressure rising; at the concentrations of κ-CG of 1.0% and 1.2%, the release rate of curcumin first showed a rising and then a falling trend with the pressure rising, reaching the maximum at 500 MPa. Chen et al. [43] developed a composite emulsion gel utilizing Freshwater mussel (*Solenaia oleivora*) protein isolate and κ-carrageenan (κ-CG) for the delivery of curcumin, achieving a maximum bioavailability of 60%. In another study, Wang et al. [44] constructed a novel food-grade Pickering emulsion for curcumin delivery using casein and chitosan, employing a transglutaminase (TGase)-catalyzed glycation method. After 2 h of simulated intestinal digestion, the final release rates of curcumin from emulsions stabilized by casein, cross-linked casein, and glycosylated casein were 51.12 ± 0.46%, 53.55 ± 0.50%, and 56.89 ± 0.31%, respectively. These results are significantly lower compared to the bioavailability observed in our experimental data. 

Yi et al. [45] found that a smaller droplet size could promote the transfer of curcumin to micelles, thereby increasing the bioavailability of curcumin. Silva et al. [46] found that the increased curcumin release rate was related to the FFA release rate. In summary, the alternation in curcumin release rate was related to the change in droplet size and FFA release rate, which was also confirmed by the results of 3.2. The particle size can affect the contact area between lipid and lipase, and the FFA release rate is associated with the formation of micromicelles. Although the control group showed a high FFA release in Figure 2, due to the accumulation of oil droplets and the absence of biomolecules, it was difficult to form a micellar phase, and the curcumin released rate was low. 

### 3.5. Microstructure

**Figure 5:**
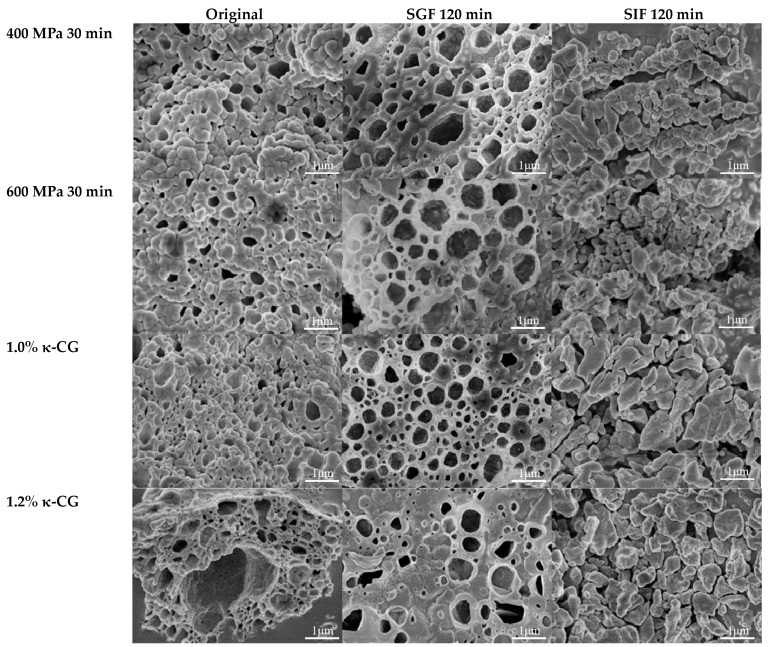
SEM images of emulsion gels prepared with different treatment pressures and κ-CG concentrations before and after digestion.

Figure 5 shows the SEM images of curcumin-loaded WPI/κ-CG gels prepared with various treatment pressures and κ-CG concentrations before digestion, after the gastric phase, and after the intestinal phase. As illustrated in the figure, the gel network structure of all emulsion gels after gastric digestion was well preserved, indicating that the structure of HPP-induced emulsion gel was not easily destroyed during gastric digestion, which might be due to the fact that WPI was not easily digested in the stomach. After 2 h of intestinal digestion, the network structure was destroyed; the curcumin dissolved in MCT was released, and needle-like curcumin crystals could be observed in the intestinal phase.

It could also be seen that with increasing pressure, a more uniform and dense network structure formed. The network structure of WPI/κ-CG gel was well preserved after gastric digestion and was destroyed to form small particles after intestinal digestion. With increasing pressure, the particles produced by emulsion gels during intestinal digestion were larger. With the rising concentration of κ-CG, the pore size of the formed gel network structure increased significantly, and the distribution became uneven, resulting in a reduction in the elasticity and chewiness of the gel and the formation of smaller particles after intestinal digestion. The emulsion gel with 1% κ-CG was more stable than the one with 1.2% κ-CG, which might be ascribed to the fact that an excess of polysaccharides would have an adverse effect on the coupling between protein and polysaccharides, ultimately leading to the destruction of the gel’s network structure. 

### 3.6. Cytotoxicity Assay

According to previous research, it can be found that the composite emulsion gel was transformed into a nano-scale emulsion after undergoing simulated gastrointestinal digestion. Although some studies have shown that nanocarriers composed of natural macromolecules were not biologically toxic [47], Penn et al. [48] found that FFA produced during the digestion process and bile salts were toxic to small intestinal epithelial cells. In addition, curcumin itself might have a destructive effect on the Caco-2 monolayer cell model because of its certain anti-cancer effects. Therefore, to clarify whether the digesta was toxic to Caco-2 cells and to guarantee the reliability of the monolayer model in subsequent transport and absorption experiments, MTT was used to detect the effects of digesta on Caco-2 cells. 

**Figure 6:**
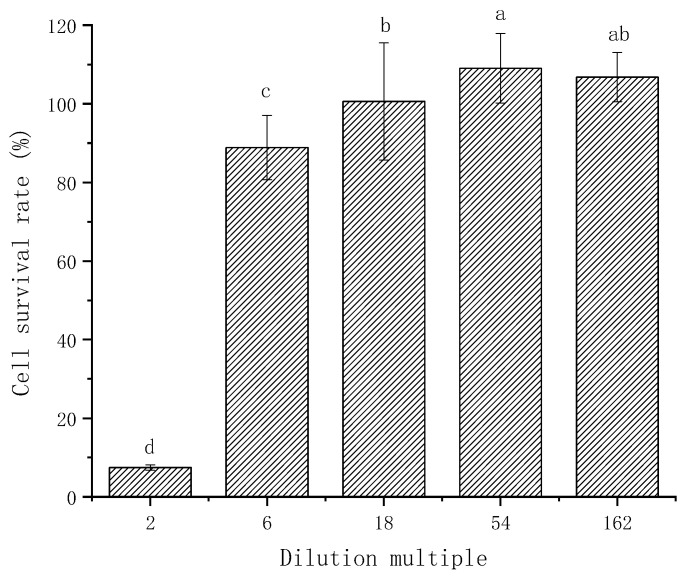
In vitro cytotoxicity of intestinal digesta at different diluted times on Caco-2 cells. Note: Different letters indicate a significant difference (*p* < 0.05, *n* = 3).

Figure 6 shows the toxicity of the digesta diluted by different multiples to Caco-2 cells. The results show that when the digest was diluted two times, the survival rate of Caco-2 cells was only 7.45 ± 0.70%, indicating that the digesta had strong toxicity and inhibitory effects on cancer cells; however, when the digesta was diluted six times, it was almost non-toxic to the cells within 2 h (that is, the cell survival rate reached more than 75%). The cell survival rate remained unaltered when the digestate was further diluted, a finding that aligns with the results reported by Yu et al. [49]. Therefore, in this experiment, the digesta diluted six times was used as the sample for the subsequent transfer and absorption experiment.

### 3.7. Cellular Curcumin Uptake

**Figure 7:**
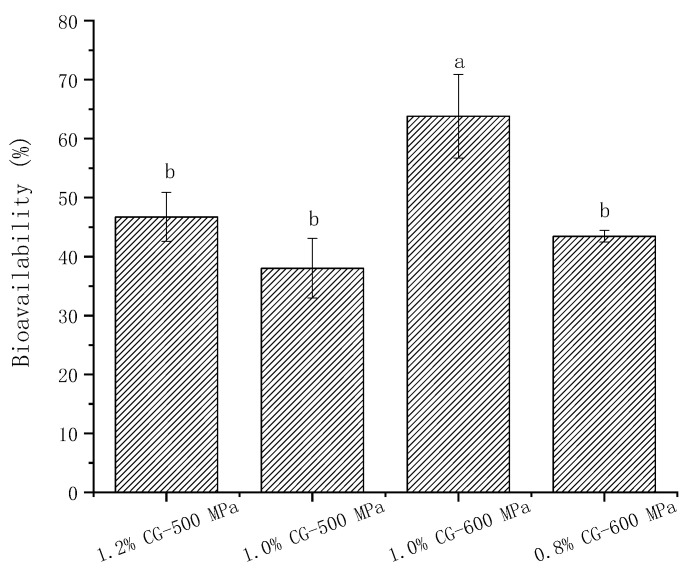
The Caco-2 cells (AP to BL) transport rate of curcumin in different emulsion gels. Note: Different letters indicate a significant difference (*p* < 0.05, *n* = 3).

According to the previous results of the curcumin release rate in the simulated gastrointestinal digestion, 0.8% κ-CG 600 MPa, 1.0% κ-CG 500 MPa, 1.0% κ-CG 600 MPa, and 1.2% κ-CG 500 MPa were selected for transport experiment. For each sample, the transfer rate (i.e., bioavailability) of curcumin in different samples through the Caco-2 cell model was determined with the results presented in Figure 7. According to the previous literature, after directly mixing free curcumin with Caco-2 cells and after 6 h of incubation, curcumin could not be detected in the cells, indicating that it was difficult for Caco-2 cells to directly take up curcumin [50]. In this experiment, after embedding biological macromolecules in an emulsion gel delivery system, the bioavailability of curcumin through Caco-2 cells was significantly improved. Among them, the bioavailability of curcumin carried by the 1.0% κ-CG 600 MPa sample, which was 63.81 ± 7.10%, was significantly higher than that of the other three samples.

Li et al. [51] designed emulsion gels based on gallic acid-modified chitosan nanoparticles (GCS NPs) by self-assembly between gelatin and Pickering emulsion. Their designed emulsion gels and beads significantly increased the cellular uptake of Cur by 61.99 % and 59.32 %, respectively. And the uptake rate of the emulsion gel was higher than that of the beads.

Taken together with the previous data on curcumin release rates, our findings demonstrate a significant increase in the bioavailability of curcumin. An increase in bioavailability translates to a higher concentration of curcumin being available to target tissues, which could lead to enhanced efficacy of the compound in potential therapeutic applications. Simultaneously, the potential reduction in curcumin dosage may mitigate the expenses related to higher amounts, and enhanced bioavailability in formulations is likely to boost patient adherence, primarily due to the decreased need for frequent administration.

## 4. Conclusions

This study assessed the efficacy of high-pressure processing (HPP)’s efficacy in enhancing curcumin’s delivery and bioaccessibility within emulsion gels, following simulated gastric and intestinal digestion. We found that increasing pressure levels and κ-carrageenan (κ-CG) concentrations significantly bolstered the stability of the curcumin delivery system. Notably, elevated κ-CG concentrations impeded proteolytic dissociation of proteins from the gel matrix, while HPP-induced emulsion gels effectively retarded free fatty acid (FFA) release and curbed curcumin release during the gastric phase.

The impact of pressure on FFA and curcumin release was dependent on κ-CG concentration: at 0.8% κ-CG, release rates escalated with pressure, whereas at 1.0% and 1.2% κ-CG, the optimal release occurred at 500 MPa. Scanning electron microscopy (SEM) revealed that higher pressures fostered a denser and more uniform gel network, which remained intact post-gastric digestion but disintegrated into larger particles following intestinal digestion, with particle size correlating positively with pressure.

Cytotoxicity assays indicated that intestinal phase digesta exhibited significant toxicity toward Caco-2 cells, highlighting potential safety concerns. Among the formulations tested, the emulsion gel with 1.0% κ-CG processed at 600 MPa demonstrated the most promising curcumin bioavailability, peaking at 63.82 ± 7.10%.

While our emulsion gels demonstrate promising results, they are not without limitations for practical application. Firstly, although high-pressure treatment bolsters the stability of the curcumin delivery system, the associated increase in pressure could potentially impact the organoleptic characteristics of the gel, including taste and appearance. Secondly, this study did not include storage experiments, which means the long-term stability of the gels remains undetermined. Thirdly, the implementation of high-pressure treatment is likely to escalate costs, necessitating further economic assessments to evaluate its feasibility. Additionally, in vivo studies would be necessary to confirm the bioavailability and efficacy of these emulsion gels in a physiological context.

These findings underscore the potential of HPP-induced emulsion gels as a delivery system to enhance curcumin’s bioaccessibility and cellular uptake, offering valuable insights for the development of more effective dietary interventions.

## Figures and Tables

**Figure A1:**
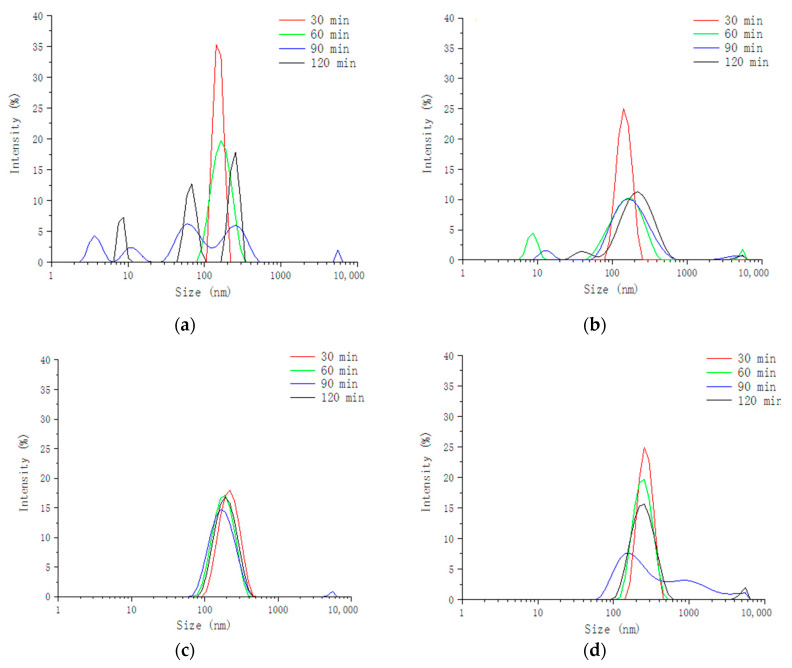

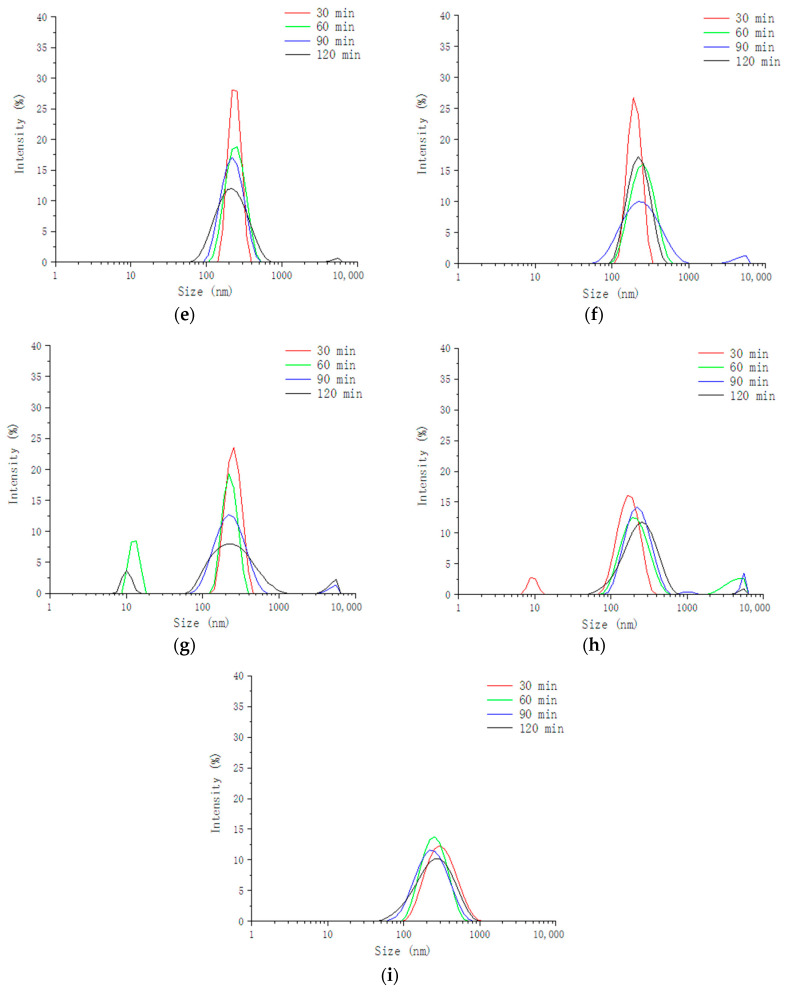
Size distribution of digesta from simulated gastric phase after different digestion times. (**a**) 0.8% κ-CG 400 MPa; (**b**) 0.8% κ-CG 500 MPa; (**c**) 0.8% κ-CG 600 MPa; (**d**) 1.0% κ-CG 400 MPa; (**e**) 1.0% κ-CG 500 MPa; (**f**) 1.0% κ-CG 600 MPa; (**g**) 1.2% κ-CG 400 MPa; (**h**) 1.2% κ-CG 500 MPa; (**i**) 1.2% κ-CG 600 MPa.

## Data Availability

The original contributions presented in the study are included in the article, further inquiries can be directed to the corresponding author.
